# O.4.3-6 Relationship between actigraphy derived physical activity, sedentary behaviours and sleep parameters in preschool children - a cross sectional study

**DOI:** 10.1093/eurpub/ckad133.192

**Published:** 2023-09-11

**Authors:** Piotr Matłosz, Justyna Wyszyńska, Artur Mazur, Jacek Tutak, Alejandro Martinez-Rodriguez, Jarosław Herbert

**Affiliations:** Institute of Physical Culture Sciences, Medical College, University of Rzeszów, Poland; Institute of Health Sciences, Medical College, University of Rzeszów, Poland; Institute of Medical Sciences, Medical College, University of Rzeszów, Poland; Department of Applied Mechanics and Robotics, The Faculty of Mechanical Engineering and Aeronautics, Rzeszów University of Technology, Poland; Analytical Chemistry, Nutrition and Food Science Department, University of Alicante, Spain; pmatlosz@ur.edu.pl; Institute of Physical Culture Sciences, Medical College, University of Rzeszów, Poland

## Abstract

**Purpose:**

The aim of the study was to explore the associations between physical activity and sedentary time with sleep parameters among preschool children.

**Methods:**

Children aged from 5 to 6 years (n = 676) attending to kindergartens were recruited, measures included accelerometer-derived 24 h activity from seven consecutive days and nights: moderate-to-vigorous physical activity (MVPA), sedentary time, sleep duration, sleep efficiency, wake after sleep onset (WASO), number of awakenings, Movement Index, Fragmentation Index and Sleep Fragmentation Index.

**Results:**

Longer time spent in MVPA was associated with higher sleep efficiency both in boys and girls. Longer time spent in sedentary behaviour was associated with lower sleep efficiency, and higher WASO, number of awakenings, Movement Index and Sleep Fragmentation Index. MVPA may be related to sleep efficiency and Sleep Fragmentation Index both in boys and girls. Among boys MVPA was inversely associated with WASO, number of awakenings and Fragmentation Index, while in girls with sleep duration, Movement Index and Sleep Fragmentation Index. Sedentary time was inversely associated with sleep efficiency and directly associated with WASO, number of awakenings, Movement Index and Sleep Fragmentation Index. Meeting recommended level of MVPA was associated with better sleep efficiency, lower WASO and number of awakenings among boys

**Conclusions:**

The sedentary time from all valid days had the strongest, negative effect on sleep-related parameters in preschool children. The results of the present study appear to suggest that sedentary time reduction may contribute more to improving sleep quality than increasing MVPA.

